# Comorbidities at MS Diagnosis and Their Association With Treatment Persistence: Real‐World Clinical Data

**DOI:** 10.1002/brb3.71253

**Published:** 2026-02-05

**Authors:** Henrik Ahvenjärvi, Ida Lund, Anne M. Portaankorva, Johanna Krüger, Mervi Ryytty

**Affiliations:** ^1^ Research Unit of Clinical Medicine, Neurology University of Oulu Oulu Finland; ^2^ Clinical Neurosciences Faculty of Medicine University of Helsinki Helsinki Finland; ^3^ Neurocenter, Neurology Oulu University Hospital Oulu Finland; ^4^ Medical Research Center Oulu University Hospital Oulu Finland

**Keywords:** comorbidity, disease activity, multiple sclerosis, psychiatric disease, treatment persistence

## Abstract

**Objectives:**

The objectives were to evaluate the prevalence of comorbidities at the time of multiple sclerosis (MS) diagnosis in a Finnish cross‐sectional cohort and to analyze whether comorbidities at diagnosis associate with clinical characteristics, treatment delays, initial disease‐modifying treatment (DMT) choice, DMT persistence, and disease activity.

**Methods:**

Patients with relapsing‐remitting MS (RRMS) were recruited during their appointments at the neurology outpatient clinic of the Oulu University Hospital, Finland, between the years 2018 and 2022. The data was gathered from the hospital medical records.

**Results:**

The study cohort consisted of 421 Finnish RRMS patients, of whom 51.8% had at least one or more comorbidity at the time of MS diagnosis. Depression (16.2%) and migraine (11.6%) were the most common comorbidities. Medium‐efficacy injectable DMTs (interferon‐β and glatiramer acetate) were associated with lower treatment persistence in patients with any comorbidity or psychiatric comorbidity during the 4‐year follow‐up. Among patients with psychiatric comorbidity, the rate of DMT discontinuation was high shortly after the DMT initiation, and the annualized relapse rate at the start of the DMT was higher compared with those without psychiatric comorbidity (1.3 [SD 0.78] vs. 1.1 [SD 0.74], *p* = 0.026).

**Conclusion:**

Comorbidities, especially psychiatric diseases, are associated with lower persistence on injectable DMTs. As comorbidities complicate the treatment of RRMS, it is crucial to identify their role from early on.

## Graphical abstract

Comorbidity was present in approximately half of the patients with relapsing‐remitting multiple sclerosis in this Finnish cross‐sectional cohort. Comorbidities, especially psychiatric diseases, were associated with lower persistence on injectable disease‐modifying treatments.

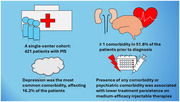

## 1 Introduction

Comorbidities are frequent among patients with multiple sclerosis (MS); as many as 50% have a co‐occurring disease at the time of diagnosis (Al‐Sakran et al. 2020). Previous studies from Finland have shown that people with MS have a higher prevalence of neurological comorbidities, as well as an increased risk of cerebrovascular diseases and type 1 diabetes (Krökki et al. 2014; Murtonen et al. 2018). Comorbidities in MS have received growing attention due to their association with diagnostic delays and adverse outcomes (Kowalec et al. 2017; Marrie et al. 2009; McKay et al. 2018; Zhang et al. 2018; Zhang et al. 2016; Lo et al. 2021). Studies have demonstrated that comorbidities can delay the initiation of disease‐modifying treatments (DMTs) (Zhang et al. 2016) and have an impact on treatment persistence (Laroni et al. 2017; Parks et al. 2021).

In this real‐world cohort study, we aimed to investigate the prevalence of comorbidities at the time of diagnosis in a Finnish cohort of patients with relapsing‐remitting MS (RRMS) and to study the associations between comorbidities and disease activity, treatment delays, certain initial MS symptoms, and DMT usage in terms of choice and persistence.

## 2 Materials and Methods

### 2.1 Data Source

**FIGURE 1:**
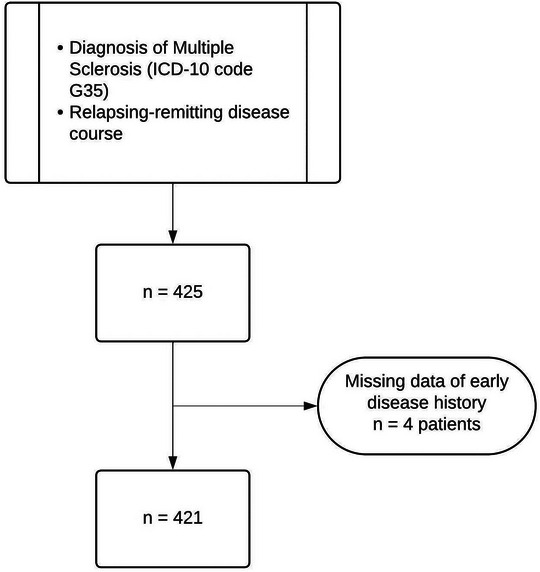
Inclusion process.

The study patients were recruited during their routine appointments at Oulu University Hospital Neurology outpatient clinic in Finland between the years 2018 and 2022. The inclusion criteria were a definite diagnosis of MS (ICD‐10 code G35) with a relapsing‐remitting disease course at the time of diagnosis (Figure 1). The diagnosis was set by a neurologist in accordance with the McDonald criteria (McDonald et al. 2001; Polman et al. 2011; Thompson et al. 2018) or Poser criteria (Poser et al. 1983) (patients diagnosed before 2001). Exclusion criteria were primary progressive or unknown disease course, or missing early disease history.

### 2.2 Variables

The following variables were collected from the hospital patient records: sex, birth date, date of disease onset, date of diagnosis, dates of DMT initiations and terminations, types of the initiated DMTs, reasons for DMT terminations, relapses, expanded disability status scale (EDSS) scores, type of symptoms at the disease onset and all the comorbidities before the date of diagnosis.

Comorbidities were gathered by a systematic review of the medical records. Identifying a comorbidity was based on a definite diagnosis made by a doctor, a diagnosis written in the patient's medical record, or regular use of certain disease‐specific medication (antihypertensive, cholesterol‐lowering, and thyroid hormone replacement medication). All the comorbidities were categorized as follows: autoimmune, psychiatric, neurological, respiratory, circulatory, dermatological, gynecological, metabolic, pain, hematological, gastrointestinal, congenital, ophthalmic, and otological disorders, and a history of cancer. The following comorbidities were considered autoimmune in nature: celiac disease, chronic uveitis, diabetes mellitus type 1, erythema nodosum, hypothyreosis or hyperthyreosis, inflammatory bowel disease (IBD; Crohn's disease or ulcerative colitis), lichen ruber planus, psoriasis, rheumatoid arthritis, sarcoidosis, and spondyloarthropathy. The complete list of comorbidities is provided in Table S1.

DMTs were categorized as medium‐efficacy injectable treatments (meINJs) (interferon‐β and glatiramer acetate), medium‐efficacy oral treatments (meORALs) (dimethyl fumarate and teriflunomide), sphingosine‐1‐phosphate receptor modulator treatments (S1PRms) (fingolimod and ponesimod), and high‐efficacy DMTs (heDMTs) (cladribine, natalizumab, ocrelizumab, alemtuzumab, rituximab, and ofatumumab). Reasons for DMT termination were categorized as follows: adverse effects, alteration of disease course, clinical ineffectiveness, pregnancy, measured drug antibodies or JC virus antibodies, patient's wish, and other reasons.

We investigated the association of comorbidities with DMT persistence by comparing how many patients with comorbidities continued to use the DMT compared with patients without comorbidities at six predefined time points: 90 days, 180 days, and yearly from 1 to 4 years after initiation.

The onset symptoms were classified based on their clinical origin within the central nervous system (CNS): (1) brainstem (double vision, specific cranial nerve and other cranial nerve symptoms); (2) cerebellum (coordination and balance disturbances); (3) spinal cord (bladder and bowel dysfunction); (4) pyramidal tract (muscle weakness and spasticity); (5) optic nerve (optic neuritis); and (6) sensory pathways (paresthesia and dysesthesia). Symptoms were considered multifocal if two or more symptoms were present, excluding sensory symptoms due to their unspecificity in terms of origin in the CNS.

Disability was evaluated with the EDSS score, ranging from 0 (normal) to 10 (death due to MS) (Kurtzke 1983). Each EDSS score was determined by a neurologist during a control visit. Baseline EDSS score was defined as the last recorded EDSS score within 1 year before the start of initial DMT.

### 2.3 Statistical Analysis

Data analysis was performed with IBM SPSS Statistics version 28.0.1.0 for Windows (IBM Corp, Armonk, NY). Partial dates were imputed as the first day or month of the year. Numerical variables were presented as means with standard deviations (SDs) for normally distributed data and as medians with interquartile ranges (IQRs) for nonparametric data. Group comparisons for categorical variables were assessed with the chi‐squared test. Two group comparisons for continuous variables were performed with Student's t‐test for normally distributed data and the Mann‐Whitney *U*‐test for nonparametric data. A *p*‐value of <0.05 was considered statistically significant.

## 3 Results

A total of 421 patients met the inclusion criteria. The majority of the patients were female (75.5%). The MS diagnoses were made between the years 1986 and 2021. The mean age at onset of symptoms was 31.0 years (SD 9.25), and the mean age at the time of diagnosis was 34.0 years (SD 9.44). The median time from MS onset to diagnosis was 11.0 months (IQR 4.00–41.0), and time from diagnosis to initial DMT start was 1.0 month (IQR 0.00–6.00). Detailed demographic information is shown in Table 1.

**TABLE 1:** Demographics and clinical characteristics.

Variable	All patients (*n* = 421)
Sex	
Female: *n* (%)	321 (75.5)
Age variables (years): mean (SD)	
Age at MS onset^a^	31.0 (25)
Age at MS diagnosis^b^	34.0 (9.44)
Age at initial DMT start^c^	35.0 (9.43)
Time delay variables (months): median [IQR]	
Time since MS onset to MS diagnosis^d^	11.0 [4.00–41.0]
Time since MS onset to initial DMT start^e^	19.0 [7.00–60.5]
Time since MS diagnosis to initial DMT start^f^	1.00 [0.00–6.00]
Relapse variables: mean (SD)	
ARR 1 year before MS diagnosis^a^	1.05 (0.64)
ARR 1 year before initial DMT start^c^	1.09 (0.75)
EDSS score at initial DMT start^g^	2.0 (1.0–3.0)
Patients on initial DMT at least 2 years; *n* (%)^h^	222 (62.5)
Initial DMT started: *n* (%)	
meINJ	288 (68.4)
meORAL	68 (16.7)
heDMT	37 (9.1)
S1PRm	6 (1.5)
Other	9 (2.2)
Onset symptom level: *n* (%)^i^	
Brainstem	73 (17.7)
Cerebellum	81 (19.7)
Pyramidal tract	76 (18.4)
Sensory pathways	192 (46.6)
Optic nerve	98 (23.8)
Spinal cord	15 (3.6)
Multifocal symptoms	43 (10.4)

Abbreviations: ARR = annualized relapse rate; DMT = disease–modifying therapy; EDSS = expanded disability status scale; heDMT = high‐efficacy disease‐modifying treatment; IQR = interquartile range; meINJ = medium‐efficacy injectable treatment; meORAL = medium‐efficacy oral treatment; S1PRm = sphingosine 1‐phosphate receptor modulator treatment; SD = standard deviation.

^a^ Missing data = 5.

^b^ Missing data = 1.

^c^ Missing data = 3.

^d^ Missing data = 6.

^e^ Missing data = 7.

^f^ Missing data = 10.

^g^ Missing data = 114.

^h^ Missing data = 53.

^i^ Missing data = 9.

### 3.1 Comorbidities

At the time of MS diagnosis, 51.8% of the patients had at least one comorbidity. Of all the patients, 23.0% had two or more comorbidities. The most prevalent comorbidities were psychiatric (16.2%), neurological (15.2%), autoimmune (13.8%), and respiratory system diseases (10.9%). Depression (12.1%) and anxiety disorder (3.6%) were the most common psychiatric disorders. Among neurological diseases, migraine (11.6%) and epilepsy (1.4%) were the most common ones. As for autoimmune diseases, thyroid disorders (8.1%), and inflammatory bowel disease (IBD) (3.6%) were the most frequent. Respiratory disorders mostly consisted of asthma (9.5%). Detailed information about comorbidities is presented in Table 2.

**TABLE 2:** Comorbidities diagnosed before MS diagnosis.

Comorbidity	All patients (*n* = 421) % (*n*)
At least one comorbidity	51.8 (218)
Psychiatric	16.2 (68)
Depression^a^	12.1 (51)
Anxiety	3.6 (15)
Other^b^	2.9 (12)
Neurological	15.2 (64)
Migraine	11.6 (49)
Epilepsy	1.4 (6)
Other^c^	2.9 (12)
Autoimmune	13.8 (58)
Hypo‐/hyperthyroidism	8.1 (34)
Inflammatory bowel disease	3.6 (15)
Other^d^	5.0 (21)
Respiratory system	10.9 (46)
Asthma	9.5 (40)
Sleep apnea	1.2 (5)
Chronic obstructive pulmonary disease	0.2 (1)
Circulatory system	7.8 (33)
Hypertension	6.9 (29)
Atrial fibrillation	0.7 (3)
Other^e^	0.7 (3)
Dermatological	3.6 (15)
Atopic dermatitis	3.3 (14)
Anetoderma of Schweninger–Buzzi	0.2 (1)
Gynecological	3.4 (11)^f^
Endometriosis	3.1 (10)^f^
Polycystic ovary syndrome	0.3 (1)^f^
Metabolic	2.9 (12)
Hyperlipidemia	2.1 (9)
Diabetes mellitus type 2	1.0 (4)
Gout	0.2 (1)
History of cancer	1.7 (7)
Pain disorder	1.7 (7)
Hematological	1.4 (6)
Hereditary coagulopathy	1.0 (4)
Other^g^	0.5 (2)
Gastrointestinal	1.4 (6)
Cholelithiasis	0.7 (3)
Other^h^	0.7 (3)
Congenital abnormality^i^	1.2 (5)
Ophthalmic	1.2 (5)
Cataract	0.5 (2)
Other^i^	0.7 (3)
Otological^k^	0.2 (1)

^a^ Missing data = 1.

^b^ Personality disorder (5), substance use disorder (3), autism spectrum disorder (1), attention‐deficit/hyperactivity disorder (1), bipolar disorder (1), psychosis (1).

^c^ History of central nervous system trauma (5), stroke (4), aneurysm (1), hydromyelia (1), narcolepsia (1).

^d^ Psoriasis (5), spondyloarthropathy (4), celiac disease (3), rheumatoid arthritis (2), type 1 diabetes (2), uveitis (2), sarcoidosis (1), lichen planus (1), erythema nodosum (1).

^e^ Cardiomyopathy (1), history of deep vein thrombosis (1), valvular heart disease (1).

^f^ Of females, *n* = 321.

^g^ Anemia (1), monoclonal gammopathy (1).

^h^ Diverticular disease (1), liver disease (2).

^i^ Talipes equinovarus (2), Arnold‐Chiari type I malformation (1), atrophic kidney (1), chromosome mosaicism (1).

^j^ Macular degeneration (2), blindness (1).

^k^ Hearing loss (1).

### 3.2 Onset Symptoms

Symptoms at onset were most frequently related to sensory pathways (46.6%), followed by optic nerve (23.8%), cerebellum (19.7%), pyramidal tract (18.4%), brainstem (17.7%), and spinal cord (3.6%). Multifocal symptoms at onset were observed in 10.4% of the patients. The data on initial symptoms were missing for nine patients. Patients with psychiatric comorbidity reported sensory symptoms more often as part of the initial symptoms than those without psychiatric disorders (58.2% vs. 44.3%, *p* = 0.045). No other statistically significant findings were observed between onset symptoms and comorbidities.

### 3.3 DMT Initiation, Choice, and Termination

DMT was initiated for 96.9% of the cohort patients. The mean age at the DMT initiation was 35.0 years (SD 9.43), and the mean EDSS score was 2.0 (IQR 1.0–3.0). Six patients were excluded from the treatment delay analysis due to starting DMT before diagnosis as part of a drug trial. The median time from onset to DMT initiation was 19.0 months (IQR 7.0–60.5). Injectable DMTs were preferred as the initial DMT, as meINJs were initiated for 70.6% of the patients and meORALs for 16.7% of the patients. S1PRms were initiated for 1.5% and heDMTs for 9.1% of the patients. The presence of a comorbidity had no association with the initial DMT choice. Between the comorbidity categories, the distribution of initial DMT choice was similar.

The most common reason for termination of the initial DMT was adverse effects (56%), followed by clinical ineffectiveness (13.2%), pregnancy (12.4%), and the patient's wish to terminate (7.9%). No differences in termination reasons were observed within comorbidity groups.

**FIGURE 2:**
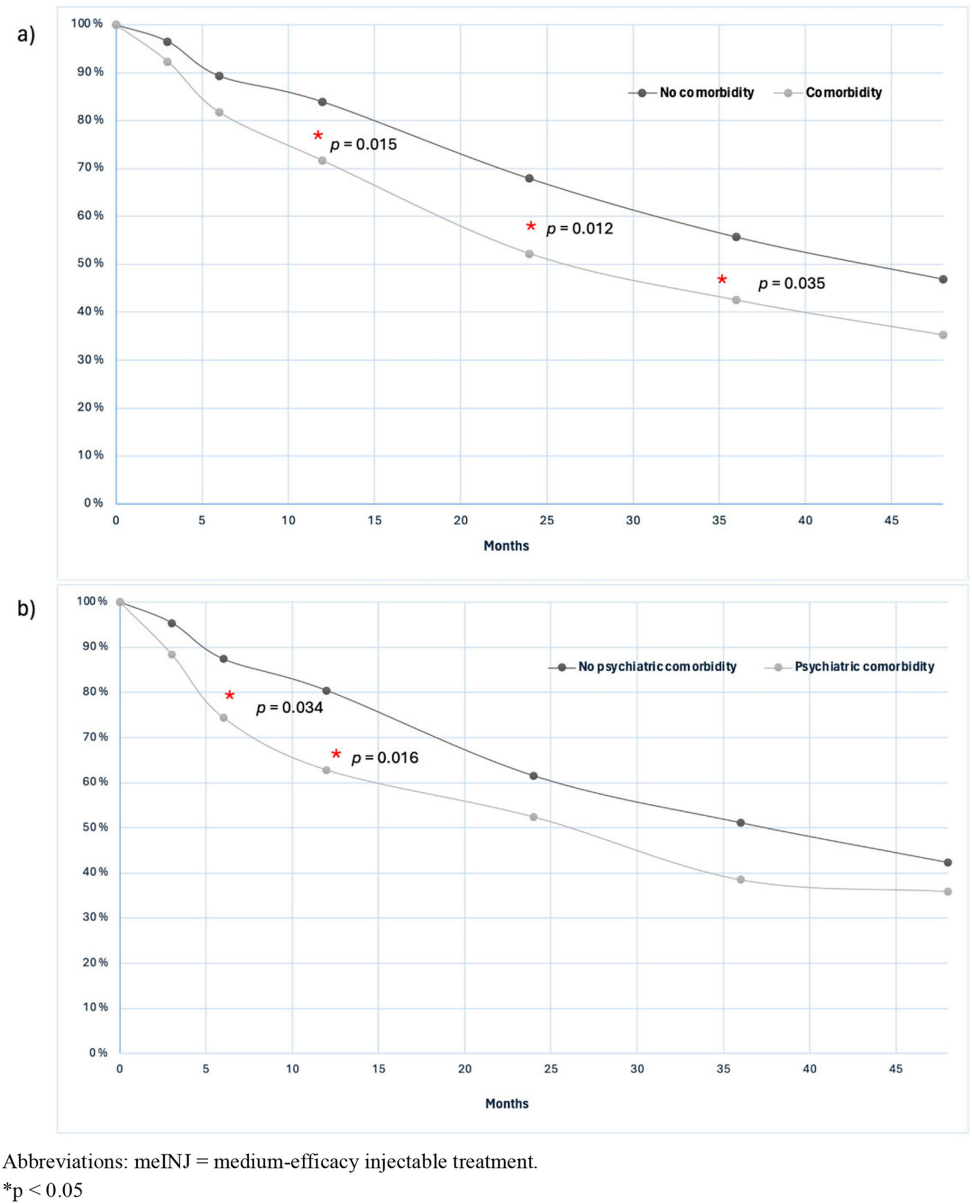
The proportion of patients on the initial meINJ with respect to time in a follow‐up time of 4 years (48 months) in the groups of (A) any comorbidity and without comorbidity, and (B) psychiatric comorbidity and without psychiatric comorbidity. meINJ = medium‐efficacy injectable treatment. *p < 0.05.

Treatment persistence was evaluated with respect to the initial DMTs during a follow‐up of 4 years. Considering all DMTs, there was a trend of lower treatment persistence among patients with any comorbidity or psychiatric comorbidity. At the 2‐year time point, patients with any comorbidity had significantly lower persistence with initial DMT than those without any comorbidity (57.1% vs. 68.2%, *p* = 0.037). However, a more detailed analysis revealed that this trend was primarily driven by meINJs, which were significantly associated with lower persistence in these comorbidity groups. Patients with any comorbidity had lower persistence with meINJs during a 4‐year follow‐up period, with significant differences at the time points of 1 year (71.6% vs. 83.9%, *p* = 0.015), 2 years (52.2% vs. 67.9%, *p* = 0.012), and 3 years (42.5% vs. 55.7%, *p* = 0.035) (Figure 2A). The reasons for meINJ discontinuation are listed in Table 3. The presence of psychiatric comorbidity was associated with lower persistence with meINJs, showing significant differences at the time points of 180 days (74.4% vs. 87.4%, *p* = 0.034) and at 1 year (62.8% vs. 80.4%, *p* = 0.016) (Figure 2B). However, after 1 year, the discontinuation rate became relatively similar to that of patients without psychiatric comorbidities. The use of meORALs or the presence of neurological or autoimmune comorbidity were not associated with lower treatment persistence (Table S2). Regarding other comorbidity groups or DMTs, the sample size was insufficient for reliable analysis.

**TABLE 3:** Reasons for terminating a medium‐efficacy injectable disease‐modifying treatment in comorbidity groups of *n* > 30.

Reasons for meINJ termination	All patients (*n* = 229)	Any comorbidity % (*n*)		Psychiatric comorbidity % (*n*)		Neurological comorbidity % (*n*)		Autoimmune comorbidity % (*n*)		Respiratory comorbidity % (*n*)		Circulatory comorbidity % (*n*)	
		Yes	No	Yes	No	Yes	No	Yes	No	Yes	No	Yes	No
Adverse effects	58.1 (133)	60.0 (69)	56.1 (64)	67.6 (25)	56.3 (108)	62.5 (20)	57.4 (113)	52.8 (19)	59.1 (114)	65.4 (17)	57.1 (116)	69.2 (9)	57.4 (124)
Alteration of disease course	0.4 (1)	0.9 (1)	0 (0)	0 (0)	0.5 (1)	3.1 (1)	0 (0)	0 (0)	0.5 (1)	0 (0)	0.5 (1)	0 (0)	0.5 (1)
Clinically ineffective	13.1 (30)	12.2 (14)	14.0 (16)	8.1 (3)	14.1 (27)	9.4 (3)	13.7 (27)	13.9 (5)	13.0 (25)	11.5 (3)	13.3 (27)	7.7 (1)	13.4 (29)
Drug antibodies	0.4 (1)	0.9 (1)	0 (0)	0 (0)	0.5 (1)	0 (0)	0.5 (1)	0 (0)	0.5 (1)	0 (0)	0.5 (1)	0 (0)	0.5 (1)
JC virus	0 (0)	0 (0)	0 (0)	0 (0)	0 (0)	0 (0)	0 (0)	0 (0)	0 (0)	0 (0)	0 (0)	0 (0)	0 (0)
Patient's wish	8.7 (20)	9.6 (11)	7.9 (9)	13.5 (5)	7.8 (15)	6.3 (2)	9.1 (18)	11.1 (4)	8.3 (16)	7.7 (2)	8.9 (18)	15.4 (2)	8.3 (18)
Pregnancy	13.5 (31)	11.3 (13)	15.8 (18)	5.4 (2)	15.1 (29)	15.6 (5)	13.2 (26)	13.9 (5)	13.5 (26)	7.7 (2)	14.3 (29)	7.7 (1)	13.9 (30)
Other	5.7 (13)	5.2 (6)	6.1 (7)	5.4 (2)	5.7 (11)	3.1 (1)	6.1 (12)	8.3 (3)	5.2 (10)	7.7 (2)	5.4 (11)	0 (0)	6.0 (13)

Abbreviation: JC = John Cunningham, meINJ = medium‐efficacy injectable treatment.

### 3.4 Disease Activity

The mean annualized relapse rate (ARR) 1 year before MS diagnosis was 1.05 (SD 0.64) and 1.09 (SD 0.75) before initial DMT start (Table 4). Among patients with psychiatric comorbidity, ARR was significantly higher a year before initial DMT start compared with those with no psychiatric comorbidity (1.3 vs. 1.1, *p* = 0.026). Two years after the initiation of the treatment, ARR was very low in all the patients, and no significant differences were observed between the groups. The median time to reach the EDSS scores of 3 and 4 after the DMT initiation was 2.9 years (IQR 0–7.4) and 5.9 years (IQR 1.2–9.2), respectively. No statistically significant differences were found with respect to reaching EDSS scores of 3 or 4 in any of the comorbidity groups.

**TABLE 4:** Treatment delays and disease activity in comorbidity groups of *n* > 30.

Variable	All patients^a^	Any comorbidity		*p*‐value	Psychiatric comorbidity		*p*‐value	Neurological comorbidity		*p*‐value	Autoimmune comorbidity		*p*‐value	Respiratory comorbidity		*p*‐value	Circulatory comorbidity		*p*‐value
		Yes	No		Yes	No		Yes	No		Yes	No		Yes	No		Yes	No	
Time from first symptoms to the first DMT, months; median [IQR]^b^	19.0 [7.00–60.5]	20.0 [7.00–59.5]	18.0 [6.25–64.8]	0.765	13.5 [5.00–58.0]	20.0 [7.00–62.5]	0.139	24.0 [7.00–58.0]	19.0 [7.00–61.8]	0.940	28.0 [8.00–102]	18.0 [7.00–57.3]	0.0600	15.0 [5.00–43.0]	20.0 [7.00–61.3]	0.310	35.5 [9.75–80.5]	19.0 [7.00–58.0]	0.0810
Time from diagnosis to the first DMT, months; median [IQR]^c^	1.0 [0.0–6.0]	1.0 [0.0–6.0]	2.0 [1.0–7.0]	0.270	1.0 [0.0–3.0]	2.0 [0.0–8.0]	0.056	1.0 [0.0–6.0]	1.0 [0.0–6.0]	0.663	2.5 [0.0–15]	1.0 [0.0–5.3]	0.364	1.0 [0.0–3.0]	1.0 [0.0–7.0]	0.164	1.0 [0.75–13]	1.0 [0.0–6.0]	0.931
ARR 1 year before DMT initiation; mean (SD)	1.1 (0.75)	1.1 (0.79)	1.0 (0.70)	0.124	1.3 (0.78)	1.1 (0.74)	**0.026**	1.1 (0.77)	1.1 (0.74)	0.791	1.2 (0.93)	0.99 (0.71)	0.212	1.3 (0.73)	1.1 (0.75)	0.083	0.83 (0.79)	1.1 (0.74)	0.0510
Annualized relapse rate 2 years since DMT initiation; median [IQR]	0.0 [0.0–0.50]	0.0 [0.0–0.50]	0.0 [0.0–0.50]	0.621	0.0 [0.0–0.3]	0.0 [0.0–0.50]	0.577	0.0 [0.0–0.0]	0.0 [0.0–0.50]	0.220	0.0 [0.0–0.50]	0.0 [0.0–0.50]	0.356	0.0 [0.0–0.38]	0.0 [0.0–0.50]	0.961	0.0 [0.0–0.0]	0.0 [0.0–0.50]	0.195
EDSS score at DMT initiation; median [IQR]^d^	2.0 [1.0–3.0]	2.0 [1.0–3.0]	2.0 [1.0–2.8]	0.661	1.0 [1.0–3.0]	2.0 [1.0–2.8]	0.514	1.0 [0.0–2.0]	2.0 [1.0–3.0]	0.053	2.0 [1.0–3.0]	2.0 [1.0–2.0]	0.261	2.0 [1.0–3.0]	2.0 [1.0–3.0]	0.770	2.0 [1.5–3.0]	2.0 [1.0–3.0]	0.113
Time to EDSS score 3 since the first DMT, years; median [IQR]^e^	2.9 [0.0–7.4]	2.7 [0.0–6.4]	3.7 [0.0–7.5]	0.651	2.5 [0.52–6.1]	3.0 [0.0–8.0]	0.438	3.4 [0.78–8.1]	2.8 [0.0–7.5]	0.919	2.5 [1.0–5.0]	3.7 [0.0–7.5]	0.605	5.5 [0.0–13.4]	2.9 [0.52–7.2]	0.819	2.8 [0.0–8.1]	2.9 [0.0–7.4]	0.624

Abbreviation: DMT = disease‐modifying treatment, ARR = annualized relapse rate, EDSS = expanded disability status scale, SD = standard deviation, IQR = interquartile range.

^a^ *n* = 405.

^b^ Missing data = 4.

^c^ Missing data = 7.

^d^ Missing data = 111.

^e^ Missing data = 326.

## 4 Discussion

In this cross‐sectional study of 421 RRMS patients, we observed that comorbidities, especially psychiatric disorders, were prevalent at the time of MS diagnosis. The presence of any comorbidity was associated with lower persistence with meINJs, with earlier treatment discontinuation observed in patients with psychiatric comorbidities. Interestingly, psychiatric comorbidities were also associated with higher ARR before DMT initiation. The study included newly diagnosed patients over an extended period (1986–2021), which explains why the majority of initial DMTs in this cohort were meDMTs. The treatment paradigm for MS has been increasingly shifting toward the initiation of high‐efficacy DMTs heDMTs, which has also been a trend in Finland during the past decade (Ahvenjärvi et al. 2025). However, despite this shift, the majority of initial DMTs prescribed in Finland in 2022 were still meDMTs (Ahvenjärvi et al. 2025), making the findings of this study highly relevant and timely.

In our study, 52% of the patients had at least one comorbidity at the time of diagnosis, which is in line with a previous study from Canada (Al‐Sakran et al. 2020). Psychiatric diseases (16.2%), particularly depression (12.1%), were the most prevalent comorbidities. The results were similar in comparison to a previous study, which reported 19% prevalence of depression or other mental health problems in the general Finnish population (Lavikainen et al. 2025). Previously reported frequencies of depression in early MS have varied from 5%–40% in studies conducted in different countries (Fromont et al. 2013; Laroni et al. 2017; Marrie et al.^2016^; Patten et al. 2017). Overall, psychiatric disorders and depression are prevalent before and after MS diagnosis (Lo et al. 2021; Marrie et al. 2016). In addition to stress and psychosocial factors related to MS diagnosis, structural changes in the brain and immune‐inflammatory factors have been suggested to predispose individuals to depression (Solaro et al. 2018).

In our study, psychiatric comorbidities were associated with a slightly higher relapse risk before DMT initiation. The significance of the result should be viewed with caution, given the small sample size and confounding factors. However, in line with our data, depression and anxiety have been associated with a higher relapse risk also in a previous study (Sparaco et al. 2022). Prior studies have identified factors that may explain the possible association between depression and disease activity in MS. MS patients with psychiatric comorbidity have been reported to have a greater burden of neuroaxonal damage and inflammation in the CNS (Tauil et al. 2021). Neuroaxonal damage markers from cerebrospinal fluid have been reported to be high in both patients with MS and in patients with depression (Novakova et al. 2017). Furthermore, MS patients with depression have a higher lesion load and greater gray matter atrophy on neuroimaging (Heitmann et al. 2022; Solaro et al. 2018). Our findings emphasize the importance of rapid initiation of a tolerable DMT, especially in MS patients with depression.

We observed an association between the presence of at least one comorbidity and earlier termination of the first DMT by 2 years after initiation. More precisely, this trend was mainly driven by meINJs. Additionally, patients with psychiatric conditions tended to terminate the initial meINJ early, as the difference compared with patients without psychiatric comorbidity was significant at 180 days after the initiation. In line with our results, the findings of a Canadian study suggest that patients with psychiatric disorders have an increased hazard for the termination of a DMT, especially in patients treated with injectable interferon‐β (Parks et al. 2021). This might be explained by a higher sensitivity among patients with psychiatric problems to injection‐related pain, needle fear, or treatment fatigue (Devonshire et al. 2011). A previous study suggested that psychiatric comorbidity is associated with increased risk of DMT discontinuation (Parks et al. 2021). An Italian study found an association between comorbidities and increased risk of terminating the first interferon‐β treatment due to intolerance (Laroni et al. 2017), while they found no association between DMT termination and specific comorbidities.

The most common reason for treatment discontinuation was adverse effects of the DMT, reported in 58% of the patients. In a previous study, a trend for an increased termination risk due to adverse effects, such as injection‐related pain or needle fear, was higher among the MS patients with a psychiatric comorbidity (Washington and Langdon 2022). Notably, interferon‐β is suspected to cause depression symptoms as side effects, but the evidence is contradictory (Alba Palé et al. 2017). We found no difference between termination reasons and the number of comorbidities or individual comorbidities. However, the small number of patients in subcohorts limited reliable analysis.

This study has many strengths. The Finnish health register system enabled the collection of comprehensive patient histories, data on prescription medicines, and systematic EDSS examinations, which have been evaluated at each patient visit for decades in our hospital. Regarding the research methods, some limitations need to be acknowledged. First, only when a comorbidity diagnosis had been given at the Oulu University Hospital could we identify an explicit diagnosis code. However, if the diagnosis had been given elsewhere, mainly in the primary health care, the diagnosis was based on the anamnestic information and data of prescription medicines. As a result, the exact times of all the diagnoses could not be reported, and it might have caused underreporting of some mild conditions, such as infrequent migraines or atopic dermatitis. However, significant underlying conditions that substantially impact the patient's health are likely to have been reliably captured. In contrast, our approach of conducting a detailed review of patient records is likely to yield more accurate results than relying solely on registry data, which in turn carries the risk of overreporting. Finally, interpretation of the findings was limited due to the cross‐sectional design of the study, preventing causal inferences.

## 5 Conclusions

Comorbidities at the time of MS diagnosis are common and impair treatment persistence, particularly considering meINJs. Therefore, the effect of possible comorbidities on treatment should be considered when choosing the first DMT. A better understanding of the effects of comorbidities is needed to support treatment decisions in patients with comorbid conditions.

## Author Contributions

**Henrik Ahvenjärvi**: conceptualization, investigation, funding acquisition, writing – original draft, methodology, validation, visualization, formal analysis. **Ida Lund**: investigation, writing – original draft. **Anne M. Portaankorva**: writing – review and editing, supervision. **Johanna Krüger**: conceptualization, writing – review and editing, supervision. **Mervi Ryytty**: conceptualization, funding acquisition, writing – review and editing, supervision.

## Funding

This study received State Research funding (AMP and JK). This work was also supported by personal grants (HA) from the Finnish Neurological Society, the Finnish Cultural Foundation, the Finnish MS Foundation, and the University of Oulu Scholarship Foundation.

## Ethics Statement

Written informed consent was obtained from all the study individuals. The research ethics committee of Northern Ostrobothnia Hospital District approved the study protocol (decision 109/2016). The study was performed according to the principles of the Declaration of Helsinki.

## Conflicts of Interest

Henrik Ahvenjärvi has received support for conference session participation and honoraria for consultant services from Novartis, and support for education sessions from Novartis and Orion. Johanna Krüger has served on the advisory board of Novartis, Nutricia, Eisai, Lilly, and Roche, and received honoraria from lectures from Bioarctic and Lilly, and received support for congress participation from Merck and Lilly. Mervi Ryytty has received honoraria for lectures, advisory boards, congress visits, or for serving as an investigator for clinical trials from AbbVie, Biogen, Merck, Novartis, Roche, and Sandoz. Ida Lund and Anne M. Portaankorva declare no conflicts of interest.

## Supporting information




**Supporting Information**: brb371253‐sup‐0001‐tableS1.docx

**Supporting Information**: brb371253‐sup‐0002‐tableS2.docx

## Data Availability

Data supporting the findings of this study cannot be made available due to confidential patient information.
