# Supplementary material for: Comorbidities at MS Diagnosis and Their Association With Treatment Persistence: Real‐World Clinical Data
Source: Brain Behav. 2026 Feb 5;16(2):e71253. doi: 10.1002/brb3.71253 (PMC12876043; doi:10.1002/brb3.71253)
Supplement: Supplementary file 1 — Supporting Information: brb371253‐sup‐0001‐tableS1.docx [file BRB3-16-e71253-s001.docx]

Supplementary Table 1. Detailed Information About the Comorbidities Diagnosed Prior to MS Diagnosis

| **Comorbidity** | **% (n)** |
| --- | --- |
| At least one comorbidity | 51.8 (218) |
| **Psychiatric** | **16.2 (68)** |
| Depression^a^ | 12.1 (51) |
| Anxiety | 3.6 (15) |
| Psychotic disorder | 1.4 (6) |
| Substance use disorder | 0.7 (3) |
| Autism spectrum disorder | 0.2 (1) |
| Bipolar disorder | 0.2 (1) |
| Hyperkinetic disorder | 0.2 (1) |
| **Neurological** | **15.2 (64)** |
| Migraine | 11.6 (49) |
| Epilepsy | 1.4 (6) |
| History of CNS trauma | 1.2 (5) |
| Cerebrovascular disease | 1.0 (4) |
| Aneurysm of cerebri media | 0.2 (1) |
| Hydromyelia | 0.2 (1) |
| Narcolepsia | 0.2 (1) |
| **Autoimmune** | **13.8 (58)** |
| Hypo-/hyperthyreosis | 8.1 (34) |
| IBD | 3.6 (15) |
| Psoriasis | 1.2 (5) |
| Spondyloartropathy | 1.0 (4) |
| Celiac disease | 0.7 (3) |
| Chronic uveitis | 0.5 (2) |
| DM type 1 | 0.5 (2) |
| Rheumatic arthritis | 0.5 (2) |
| Erythema nodosum | 0.2 (1) |
| Lichen ruber planus | 0.2 (1) |
| Sarcoidosis | 0.2 (1) |
| **Respiratory system** | **10.9 (46)** |
| Asthma | 9.5 (40) |
| Sleep apnea | 1.2 (5) |
| COPD | 0.2 (1) |
| **Circulatory system** | **7.8 (33)** |
| Hypertension | 6.9 (29) |
| Atrial fibrillation | 0.7 (3) |
| Cardiomyopathy | 0.2 (1) |
| History of vascular occlusion | 0.2 (1) |
| Valvular heart disease | 0.2 (1) |
| **Dermatological** | **3.6 (15)** |
| Atopic dermatitis | 3.3 (14) |
| Other localized connective tissue disorder | 0.2 (1) |
| **Gynecological** | **3.4 (12)^b^** |
| Endometriosis | 3.1 (11) |
| Polycystic ovary syndrome | 0.3 (1) |
| **Metabolic** | **2.9 (12)** |
| Hyperlipidemia | 2.1 (9) |
| Diabetes mellitus type 2 | 1.0 (4) |
| Gout | 0.2 (1) |
| **History of cancer** | **1.7 (7)** |
| **Pain disorder** | **1.7 (7)** |
| Chronic pain disorder | 1.2 (5) |
| Fibromyalgia | 0.5 (2) |
| **Hematological** | **1.4 (6)** |
| Hereditary coagulopathy | 1.0 (4) |
| Hereditary anemia | 0.2 (1) |
| Monoclonal gammopathy | 0.2 (1) |
| **Gastrointestinal** | **1.4 (6)** |
| Cholelithiasis | 0.7 (3) |
| Liver disease | 0.5 (2) |
| Diverticular disease | 0.2 (1) |
| **Congenital abnormality** | **1.2 (5)** |
| Congenital malformation of the musculoskeletal system | 0.5 (2) |
| Congenital malformation of the CNS | 0.2 (1) |
| Congenital malformation of the urinary system | 0.2 (1) |
| Chromosomal abnormality | 0.2 (1) |
| **Ophthalmic** | **1.2 (5)** |
| Cataract | 0.5 (2) |
| Macular degeneration | 0.5 (2) |
| Blindness | 0.2 (1) |
| **Otological** | **0.2 (1)** |
| Hearing loss | 0.2 (1) |

^a^Missing data = 1
^b^Of females = 321
