# Supplementary material for: Comorbidities at MS Diagnosis and Their Association With Treatment Persistence: Real‐World Clinical Data
Source: Brain Behav. 2026 Feb 5;16(2):e71253. doi: 10.1002/brb3.71253 (PMC12876043; doi:10.1002/brb3.71253)
Supplement: Supplementary file 2 — Supporting Information: brb371253‐sup‐0002‐tableS2.docx [file BRB3-16-e71253-s002.docx]

Supplementary Table 2. Persistence of Disease-Modifying Treatments (DMTs) in Patients with Comorbidities with Respect to Time.

|  | **90 days** | | | **180 days** | | | **1 year** | | | **2 years** | | | **3 years** | | | **4 years** | | |
| --- | --- | --- | --- | --- | --- | --- | --- | --- | --- | --- | --- | --- | --- | --- | --- | --- | --- | --- |
| **Presence of comorbidity** | **Yes, % (n)** | **No, % (n)** | **p value** | **Yes, % (n)** | **No, % (n)** | **p value** | **Yes, % (n)** | **No, % (n)** | **p value** | **Yes, % (n)** | **No, % (n)** | **p value** | **Yes, % (n)** | **No, % (n)** | **p value** | **Yes, % (n)** | **No, % (n)** | **p value** |
| **Any** |  |  |  |  |  |  |  |  |  |  |  |  |  |  |  |  |  |  |
| All DMTs | 92.8 (192) | 95.4 (186) | 0.298 | 85.4 (176) | 89.1 (172) | 0.296 | 76.3 (151) | 83.8 (155) | 0.075 | 57.1 (104) | 68.2 (118) | 0.037* | 44.6 (70) | 55.5 (91) | 0.058 | 35.9 (52) | 46.2 (72) | 0.079 |
| meINJs | 92.3 (132) | 96.5 (136) | 0.197 | 81.7 (116) | 89.3 (125) | 0.091 | 71.6 (101) | 83.9 (115) | 0.015* | 52.2 (70) | 67.9 (91) | 0.012* | 42.5 (54) | 55.7 (73) | 0.035* | 35.2 (43) | 46.9 (61) | 0.073 |
| meORALs | 89.2 (33) | 90.3 (28) | 1.00 | 89.2 (33) | 90.3 (28) | 1.00 | 82.9 (29) | 85.7 (24) | 1.00 | 64.5 (20) | 75.0 (18) | 0.558 | 58.3 (14) | 68.2 (15) | 0.552 | 47.1 (8) | 64.7 (11) | 0.491 |
| **Psychiatric** |  |  |  |  |  |  |  |  |  |  |  |  |  |  |  |  |  |  |
| All DMTs | 89.2 (58) | 95.0 (320) | 0.086 | 80.0 (52) | 88.6 (296) | 0.067 | 71.4 (45) | 81.6 (261) | 0.084 | 58.9 (33) | 63.2 (189) | 0.551 | 43.8 (21) | 51.3 (140) | 0.352 | 35.6 (16) | 42.2 (108) | 0.418 |
| meINJs | 88.4 (38) | 95.4 (230) | 0.076 | 74.4 (32) | 87.4 (209) | 0.034* | 62.8 (27) | 80.4 (189) | 0.016* | 52.4 (22) | 61.5 (139) | 0.305 | 38.5 (15) | 51.1 (112) | 0.166 | 35.9 (14) | 42.3 (90) | 0.485 |
| meORALs | 84.6 (11) | 90.9 (50) | 0.611 | 84.6 (11) | 90.9 (50) | 0.611 | 84.6 (11) | 84.0 (42) | 1.00 | 70.0 (7) | 68.9 (31) | 1.00 | 66.7 (6) | 62.2 (23) | 1.00 | 33.3 (2) | 60.7 (17) | 0.370 |
| **Neurological** |  |  |  |  |  |  |  |  |  |  |  |  |  |  |  |  |  |  |
| All DMTs | 91.7 (55) | 94.4 (323) | 0.554 | 91.5 (54) | 86.5 (294) | 0.305 | 80.4 (45) | 79.8 (261) | 1.00 | 67.3 (35) | 61.7 (187) | 0.536 | 53.2 (25) | 49.6 (136) | 0.753 | 50.0 (22) | 39.7 (102) | 0.246 |
| meINJs | 90.2 (37) | 95.1 (231) | 0.262 | 90.0 (36) | 84.7 (205) | 0.474 | 75.0 (30) | 78.2 (186) | 0.683 | 61.5 (24) | 59.8 (137) | 0.862 | 45.9 (17) | 49.8 (110) | 0.724 | 42.9 (15) | 41.0 (89) | 0.855 |
| meORALs | 92.3 (12) | 89.1 (49) | 1.00 | 92.3 (12) | 89.1 (49) | 1.00 | 91.7 (11) | 82.4 (42) | 0.671 | 90.9 (10) | 63.6 (28) | 0.143 | 88.9 (8) | 56.8 (21) | 0.124 | 87.5 (7) | 46.2 (12) | 0.053 |
| **Autoimmune** |  |  |  |  |  |  |  |  |  |  |  |  |  |  |  |  |  |  |
| All DMTs | 93.0 (53) | 94.2 (325) | 0.761 | 82.5 (47) | 88.0 (301) | 0.282 | 75.0 (42) | 80.7 (264) | 0.366 | 56.6 (30) | 63.6 (192) | 0.358 | 45.8 (22) | 50.9 (139) | 0.535 | 35.7 (15) | 42.1 (109) | 0.501 |
| meINJs | 95.2 (40) | 94.2 (228) | 1.00 | 81.0 (34) | 86.3 (207) | 0.476 | 73.8 (31) | 78.4 (185) | 0.547 | 53.7 (22) | 61.2 (139) | 0.389 | 42.1 (16) | 50.5 (111) | 0.383 | 35.1 (13) | 42.3 (91) | 0.472 |
| meORALs | 80.0 (8) | 91.4 (53) | 0.582 | 80.0 (8) | 91.4 (53) | 0.582 | 66.7 (6) | 87.0 (47) | 0.145 | 55.6 (5) | 71.7 (33) | 0.435 | 50.0 (4) | 65.8 (25) | 0.443 | 33.3 (1) | 58.1 (18) | 0.571 |

Abbreviations: DMT = disease-modifying treatment, meINJ = medium-efficacy injectable treatment, meORAL = medium-efficacy oral treatment
*p < 0.05
